# STAHD: a scalable and accurate method to detect spatial domains in high-resolution spatial transcriptomics data

**DOI:** 10.1093/bioinformatics/btaf619

**Published:** 2025-11-10

**Authors:** Zhihua Du, Di Wang, Qiyi Chen, Yuehua Ou, Xinlei Huang, Xiang Zhou, Xubin Zheng

**Affiliations:** College of Computer Science and Software Engineering, ShenZhen University, Shenzhen, Guangdong, 518000, China; College of Computer Science and Software Engineering, ShenZhen University, Shenzhen, Guangdong, 518000, China; Dongguan Key Laboratory for AI and Dynamical Systems, School of Computing and Information Technology, Great Bay University, Dongguan, Guangdong, 523000, China; College of Computer Science and Software Engineering, ShenZhen University, Shenzhen, Guangdong, 518000, China; College of Computer Science and Software Engineering, ShenZhen University, Shenzhen, Guangdong, 518000, China; Dongguan Key Laboratory for AI and Dynamical Systems, School of Computing and Information Technology, Great Bay University, Dongguan, Guangdong, 523000, China; Guangdong Institute of Intelligence Science and Technology, Hengqin, Zhuhai, Guangdong, 519031, China; Dongguan Key Laboratory for AI and Dynamical Systems, School of Computing and Information Technology, Great Bay University, Dongguan, Guangdong, 523000, China

## Abstract

**Motivation:**

Spatial transcriptomics (ST) enables the study of spatial heterogeneity in tissues. However, current methods struggle with large-scale, high-resolution data, leading to reduced efficiency and accuracy in detecting spatial domains. A scalable, precise solution is urgently needed.

**Results:**

We present STAHD, a scalable and efficient framework for spatial domain detection in ST data. Combining a graph attention autoencoder with multilevel *k*-way graph partitioning, STAHD decomposes large graphs into compact subgraphs and generates low-dimensional embeddings. This improves computational efficiency and clustering accuracy. Benchmarks on human and mouse datasets show STAHD outperforms existing methods and accurately reveals spatially distinct tumor microenvironments and functional regions.

**Availability and implementation:**

Source code and data are available at: https://github.com/Little-Eel/STAHD.

## 1 Introduction

Next-generation spatially resolved transcriptomics technologies are rapidly advancing toward high-resolution platforms such as 10x Visium-HD (Oliveira *et al.* 2025), 10x Xenium (Xenium In Situ Platform 2024), and CosMx (He *et al.* 2022), which enable subcellular resolution. This advance has led to a substantial increase in data volume, with single-section datasets reaching millions of capture spots in recent studies (Kukanja *et al.* 2024, Liu *et al.* 2025, Oliveira *et al.* 2025). As a result, new computational challenges arise in accurately delineating spatial tissue architecture and identifying spatially specific gene expression patterns (Huang *et al.* 2024, Xie *et al.* 2025).

Existing spatial domain detection methods, such as SEDR (Xu *et al.* 2024), STAGATE (Dong and Zhang 2022), SpaceFlow (Ren *et al.* 2022), and GraphST (Long *et al.* 2023), face challenges in scalability and clustering accuracy due to the rapidly increasing size of high-resolution ST datasets (Covert *et al.* 2023, Zhu *et al.* 2024). They typically require loading the entire spatial neighbor graph into memory, resulting in high computational burden when processing million-cell-scale data. Furthermore, existing approaches have limited the capacity in modeling complex spatial interactions and technical noise, especially at domain boundaries. These limitations severely constrain the potential applications of high-resolution ST data in dissecting tissue heterogeneity (Xie *et al.* 2024, Zheng *et al.* 2024, 2021, Meng *et al.* 2024).

**Figure 1.**
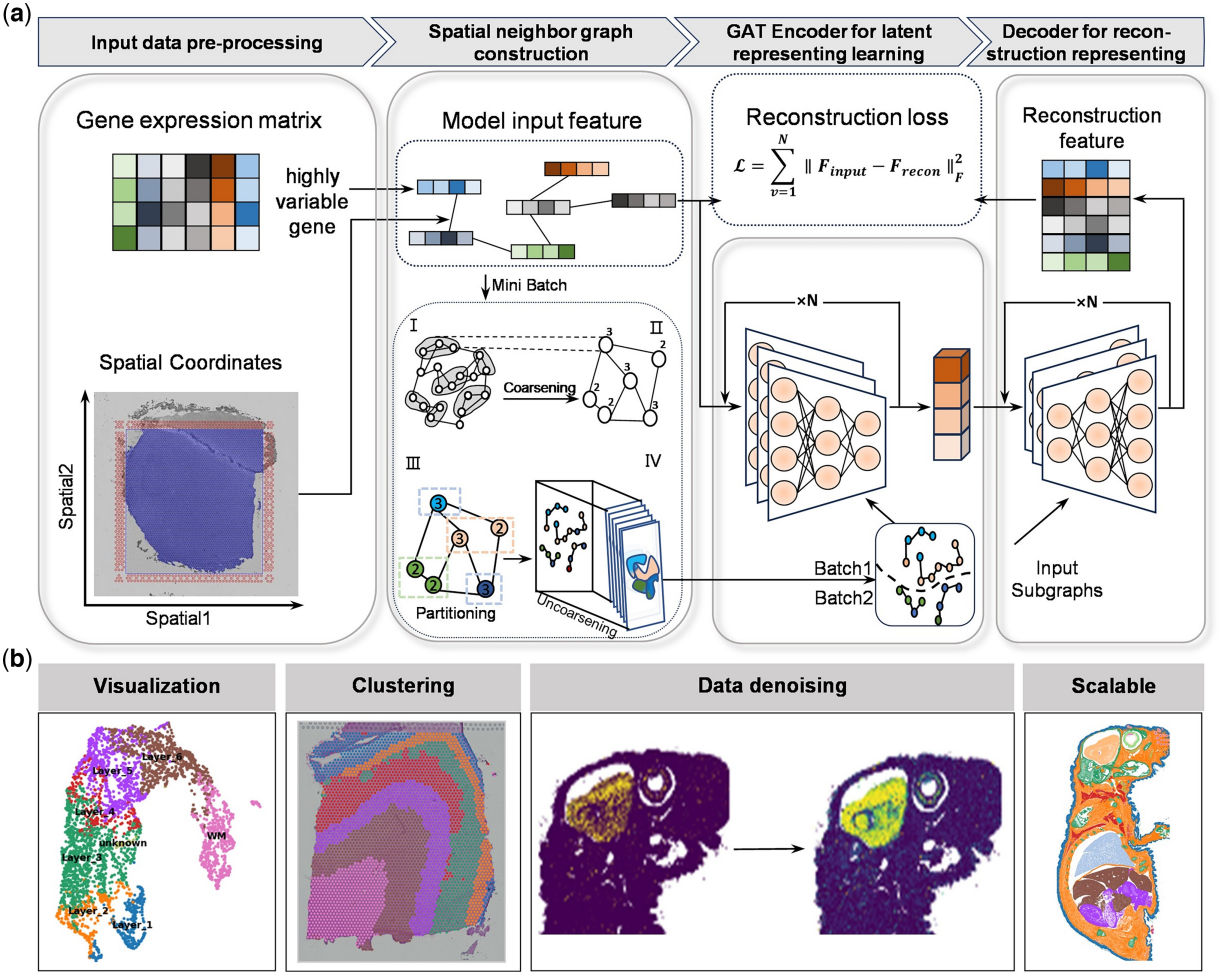
Overview of STAHD. (a) Schematic workflow of STAHD. Inputs: a gene expression matrix and spatial coordinates. Processing steps: (i) normalization and selection of highly variable genes; (ii) construction of a spatial neighbor graph; (iii) multi-level *k*-way partitioning with coarsening and refinement to divide the graph into subgraphs; (iv) graph attention autoencoder for mini-batch training and latent representation learning; and (v) reconstruction of input features and optimization via reconstruction loss. Outputs: spatially informed low-dimensional embeddings that preserve both gene expression and spatial structure while reducing computational overhead. (b) STAHD generates low-dimensional embeddings for visualization and clustering of high-resolution spatial transcriptomic data, while performing data denoising and enabling scalable analysis on tissue sections containing millions of spatial spots.

To address these challenges, we propose STAHD, a scalable algorithm for spatial domain detection in high-resolution ST data (Fig. 1). STAHD employs a graph attention autoencoder to integrate spatial coordinates and gene expression, learning low-dimensional embeddings for spatial domain identification. An attention mechanism adaptively models spatial similarity, particularly at domain boundaries. To reduce computational burden, we leverage a multilevel *k*-way graph partitioning strategy to recursively divide large graphs into compact subgraphs for independent training, enabling efficient processing of million-cell-scale datasets. STAHD was validated on diverse ST datasets from multiple platforms including 10x Visium (DLPFC), Visium-HD (human tonsil and human breast cancer), Xenium (whole adult mouse), and CosMx (human lymph node), and consistently outperformed existing methods in distinguishing tissue structures and computational resource consuming.

## 2 Materials and methods

### 2.1 Datasets

To train and evaluate the applicability and performance of STAHD across diverse tissue architectures and spatial resolutions, five high-resolution spatial transcriptomics (ST) datasets generated from different sequencing platforms and tissue types were used in this study. Details and the preprocessing procedures of the datasets can be found in Supplementary File 1, available as supplementary data at *Bioinformatics* online.

### 2.2 Spatial neighborhood graph construction

Based on the spatial coordinates of all capture spots, pairwise Euclidean distances were computed to quantify spatial proximity. Two spots were considered spatial neighbors if the distance between them was smaller than a predefined threshold *r*, which can be adjusted accordingly. According to these spatial neighborhood relationships, an undirected spatial neighbor graph was constructed and represented by an adjacency matrix *A*, where $A_{ij}$ = 1 if an edge exists between spot *i* and spot *j*, and $A_{ij}=0$ otherwise. Additionally, self-loop edges were added to each spot to preserve the self-information of each node during subsequent graph neural network computations. To ensure consistency across datasets with different spatial resolutions, STAHD employs an adaptive radius-selection module that automatically determines the spatial radius (*r*) based on each dataset’s resolution. The spatial radius *r* represents the physical distance used to define neighborhood relationships among spots and determines the number of neighboring spots, we evaluated model performance with respect to the average neighbor count, which provides a more interpretable measure of the effective neighborhood size. Sensitivity analysis on the DLPFC dataset (Fig. 1c and d, available as supplementary data at *Bioinformatics* online) showed that clustering performance was optimal when each spot had approximately 10–30 neighbors, achieving a balance between capturing spatial context and reducing noise. For other datasets, *r* was automatically adjusted to maintain this neighbor range, ensuring biologically meaningful and consistent spatial connectivity across spatial platforms. This strategy follows the design adopted by STAligner (Zhou *et al.* 2023), ensuring cross-platform consistency in physical distance scaling.

### 2.3 Multilevel *k*-way graph partitioning strategy

To enable efficient analysis of large-scale ST graph data, this study leverages a multilevel k-way graph partitioning strategy with graph neural networks. This approach progressively simplifies the original large graph, performs an initial partitioning, and subsequently refines the partitions, thereby decomposing million-node graphs into multiple structurally compact and load-balanced subgraphs for parallel training. This significantly reduces the computational complexity per training iteration. Specifically, let the original graph be denoted as $G_{0}=(V_{0},E_{0},w)$, where $V_{0}$ is the set of nodes with size $|V_{0}|=N$, and $E_{0}$ is the set of unweighted edges. The edge weight function is defined as:


(1) w(e)={1, if edge e exists0, otherwise   

All nodes were initially assigned a uniform weight of 1, i.e. w(v)=1 for all $v\in V_{0}$. To address the computational challenges posed by large-scale graph partitioning, this method consists of three sequential stages: graph coarsening, initial partitioning, and multilevel refinement through uncoarsening.

#### 2.3.1 Graph coarsening

In the multilevel graph partitioning framework, the original graph $G_{0}=(V_{0},E_{0})$ is progressively compressed into a series of coarsened graphs $G_{1},G_{2},\ldots ,G_{m}$ with decreasing sizes. In each coarsening step, the algorithm randomly iterates over all unmatched nodes in the current graph. For each unmatched node u, one of its unmatched neighboring nodes v is randomly selected, and the two are merged into a supernode denoted as C(u,v). If no unmatched neighbor exists for node u, it is retained as an individual super node. The weight of each super node is defined as the sum of the weights of its constituent nodes:


(2) $$w(C)=\sum_{v\in C}\;w(v)\;$$

where C denotes the super node and v represents the original nodes contained within it. All original nodes are initially assigned a weight of 1, i.e. w(v)=1. The edges between super nodes are determined based on the connections among the original nodes. Specifically, if any pair of original nodes belonging to two different supernodes are connected in the original graph, an edge is established between the corresponding super nodes in the coarsened graph.

In standard multilevel graph partitioning methods, a coarsening termination threshold $N_{c}$ is typically predefined. The coarsening process stops when the number of nodes in the coarsened graph $\left|V_{i}\right|$ drops below this threshold, at which point initial partitioning is performed. The value of $N_{c}$ is generally chosen based on the target number of partitions k, computational resources, or a tradeoff between partitioning speed and accuracy, with typical values ranging from $100\leq N_{c}\leq 1000$.

In contrast, the METIS-based multilevel partitioning strategy adopted in this study employs an adaptive heuristic rule to dynamically determine the termination point of coarsening without explicitly setting a fixed $N_{c}$. Specifically, the algorithm automatically decides the optimal coarsened graph scale based on the graph size after each coarsening iteration, the trend of cut-edge reduction, and partition quality estimations. This ensures that the final coarse graph is sufficiently small to improve initial partitioning efficiency while avoiding excessive coarsening that would otherwise degrade partitioning accuracy.

#### 2.3.2 Initial partitioning

Initial partitioning is performed on the coarsened graph $G_{m}$. Since the edges in the graph are unweighted (with all edge weights set to 1), the partitioning phase no longer relies on edge weights or extended subgraphs, but instead adopts a degree-based greedy growing strategy. Specifically, the algorithm begins from multiple randomly selected seed supernodes and iteratively absorbs their unpartitioned neighboring supernodes into the current subgraph. Priority is given to neighboring super nodes with higher degrees to enhance intra-subgraph connectivity and minimize the number of cut edges. To ensure balanced partition sizes, the number of partitions is determined as:


(3) numparts=Nbatchsize×10

where the batch size was set to 256 by default, but can be adjusted according to the requirements of different ST platforms and resolutions. Accordingly, the approximate capacity limit for a single subgraph is:


(4) cap=Nnum_parts=N/Nbatch_size×10≈25610=25.6

Once the number of super nodes in a subgraph reaches this capacity, expansion for that subgraph stops, and the algorithm proceeds to build the next subgraph. After initial partitioning, a structurally compact and load-balanced subgraph division scheme is obtained.

#### 2.3.3 Uncoarsening and refinement

After initial partitioning on the coarsened graph $G_{m}$, the partitioning results are progressively projected back to the original graph $G_{0}$ through a series of uncoarsening steps. In each step from $G_{i}$ to $G_{i-1}$, the subgraph label of each supernode is propagated to all of its constituent nodes:


(5) label(v)=label(C(v)), ∀v∈C

To further improve partitioning quality, a local refinement procedure is applied to boundary nodes between subgraphs. Specifically, for each candidate boundary node v, the number of cut edges connected to other subgraphs before the potential migration is computed and denoted as ${\text{cut}}_{\text{before}}(v)$. Then, the number of cut edges is recalculated under the hypothetical scenario in which node v is migrated to a neighboring subgraph $V_{j}$, denoted as ${\text{cut}}_{\text{after}}(v)$. The gain in cut reduction achieved by this migration is defined as:


(6) g(v)=cutbefore(v)-cutafter(v)

If g(v)>0, indicating that the migration would reduce the total number of cut edges and improve subgraph compactness, the algorithm further checks whether the target subgraph $V_{j}$ satisfies the capacity constraint:


(7) |Vj|+1≤cap

If both conditions are met, the migration operation is performed: node v is moved from its current subgraph to the target subgraph $V_{j}$, and its label is updated accordingly. This process iterates over all eligible boundary nodes, sequentially applying migrations to reduce inter-subgraph cut edges while maintaining balanced partition loads. Finally, this results in a high-quality partitioning on the original graph $G_{0}$ with compact, well-defined subgraph boundaries and balanced sizes, providing an optimized structural foundation for subsequent graph neural network training.

### 2.4 Graph attention autoencoder

We partition the original graph into several structurally compact subgraphs with balanced node counts, serving as the basic units for model training. During training, a mini-batch loading strategy is employed, where each batch consists of multiple subgraphs. The default batch size is set to 10 (a tunable hyperparameter) to balance computational load per iteration and model convergence speed.

The encoder takes the gene expression matrix $X^{\left(b\right)}\in R^{n_{b}\times d}$ and the corresponding spatial adjacency matrix for each subgraph as input, where $n_{b}$ denotes the number of nodes in subgraph b, and d is the dimensionality of gene expression features. The encoder comprises L graph attention layers that iteratively extract node-level latent representations. The representation of node i at the kth layer (k=1,2,…,L−1) is computed as:


(8) Zi(k)=σ(∑j∈Ni(b) αij(k)W(k)Zj(k−1))

where $Z_{i}^{\left(0\right)}=X_{i}^{\left(b\right)}$ is the initial feature of node i, $W^{\left(k\right)}$ is the trainable weight matrix at the kth layer, σ(⋅) is a nonlinear activation function, and $N_{i}^{\left(b\right)}$ denotes the neighborhood of node i (including itself). The attention coefficient $\alpha_{ij}^{\left(k\right)}$ adaptively measures the influence of neighboring node j on node i, computed as:


(9) αij(k)=exp⁡(eij(k))∑ι∈Ni(b) exp⁡(eil(k))

where $e_{ij}^{\left(k\right)}$ represents the association score between node i and its neighbor j, defined as:


(10) eij(k)={Sigmoid((a(k))T[W(k)Zi(k−1)∥W(k)Zj(k−1)]), if Aij=10, otherwise

Here, ∥ denotes vector concatenation, $a^{\left(k\right)}$ is a learnable attention vector at layer k, and ⊤ indicates matrix transposition. The final Lth layer omits the attention mechanism and directly computes the latent embedding as:


(11) Zi(L)=σ(W(L)Zi(L−1))

The output $Z_{i}^{\left(L\right)}$ is treated as the final latent representation of node i for downstream tasks.

The decoder is symmetric to the encoder, reversing the encoding process to reconstruct the original gene expression features. At each layer k=1,2,…,L−1, the reconstructed representation of node i is:


(12) Z^i(k)=∑j∈Ni(b) α^ij(k)σ(W^(k+1)Z^j(k+1))

where ${\hat{W}}^{\left(k+1\right)}={\left(W^{\left(k+1\right)}\right)}^{T}$ and ${\hat{\alpha }}_{ij}^{\left(k\right)}=\alpha_{ij}^{\left(k+1\right)}$. The final layer reconstructs the gene expression features as:


(13) Z^i(L)=σ(W^(1)Z^i(1))

The output ${\hat{Z}}_{i}^{\left(L\right)}$ represents the reconstructed gene expression profile for node i.

To ensure the latent embedding preserves the information of the original gene expression data, a graph attention autoencoder structure is adopted, with the reconstruction error of the gene expression matrix as the primary optimization objective. Given the original input expression matrix $X^{\left(b\right)}\in R^{n_{b}\times d}$ and the subgraph structure $G_{b}$, the encoder produces the latent representation $Z^{\left(b\right)}\in R^{n_{b}\times d}$, and the decoder reconstructs it as:


(14) X^(b)=Z(b)Wd+b

where $W_{d}\in R^{d\times p}$ and $b\in R^{p}$ are the decoder parameters, with d=30 as the embedding dimensionality. The training objective minimizes the mean squared reconstruction error (MSE):


(15) Lrecon=∑b=1m X(b)-X^(b)F2

where $∥\cdot {∥}_{F}$ denotes the Frobenius norm. To prevent gradient explosion, gradient clipping is applied during training, limiting the gradient norm to 5. A three-layer network with an embedding dimension of 64 was implemented and optimized using the Adam optimizer with a learning rate of 0.001 and a weight decay of 1e−4. The model was trained for 1000 epochs and executed on an NVIDIA GPU.

For comparison, we adopted the official implementations of the benchmark methods [SEDR, STAGATE, SpaceFlow, GraphST, and PAST (Li *et al.* 2023)] and followed their recommended or default hyperparameter settings. All methods were applied to the same preprocessed datasets under identical experimental conditions to ensure a fair and reproducible comparison. All experiments were performed on a Linux server (kernel version 6.8.0) equipped with 40 CPU cores, 503 GB of system RAM, and two NVIDIA RTX A5000 GPUs (each with 24 GB memory).

## 3 Results

### 3.1 Overview of STAHD

STAHD takes the normalized gene expression matrix and spatial coordinates of tissue sections as input, and learns latent representations by modeling both transcriptional profiles and spatial information. For each ST slice, STAHD first constructs spatial neighbor graph based on the spatial coordinates of all capture spots. This process involves computing pairwise Euclidean distances, determining neighborhood relationships using a predefined distance threshold, and subsequently constructing adjacency matrix. To enhance scalability for large-scale ST datasets, STAHD recursively divides the original graph into several locally compact and computationally balanced subgraphs as inputs using multilevel *k*-way graph partitioning. This ensures that local neighborhood structures are preserved within each partition while enabling independent and parallel training of subgraphs.

Subsequently, a graph attention autoencoder is employed to independently encode each subgraph. The encoded low-dimensional embeddings are then decoded to recover the original input features, with the reconstruction error serving as the model’s optimization objective. By minimizing the reconstruction loss, STAHD effectively captures low-dimensional representations that preserve both spatial structural context and transcriptional feature information, providing a reliable foundation for downstream analysis.

Finally, STAHD enables multiple functionalities, including spatial domain identification and visualization, data denoising, and scalable analysis for large-scale ST datasets.

### 3.2 Comparison of STAHD with four methods on 10x Visium DLPFC slice for spatial clustering

**Figure 2.**
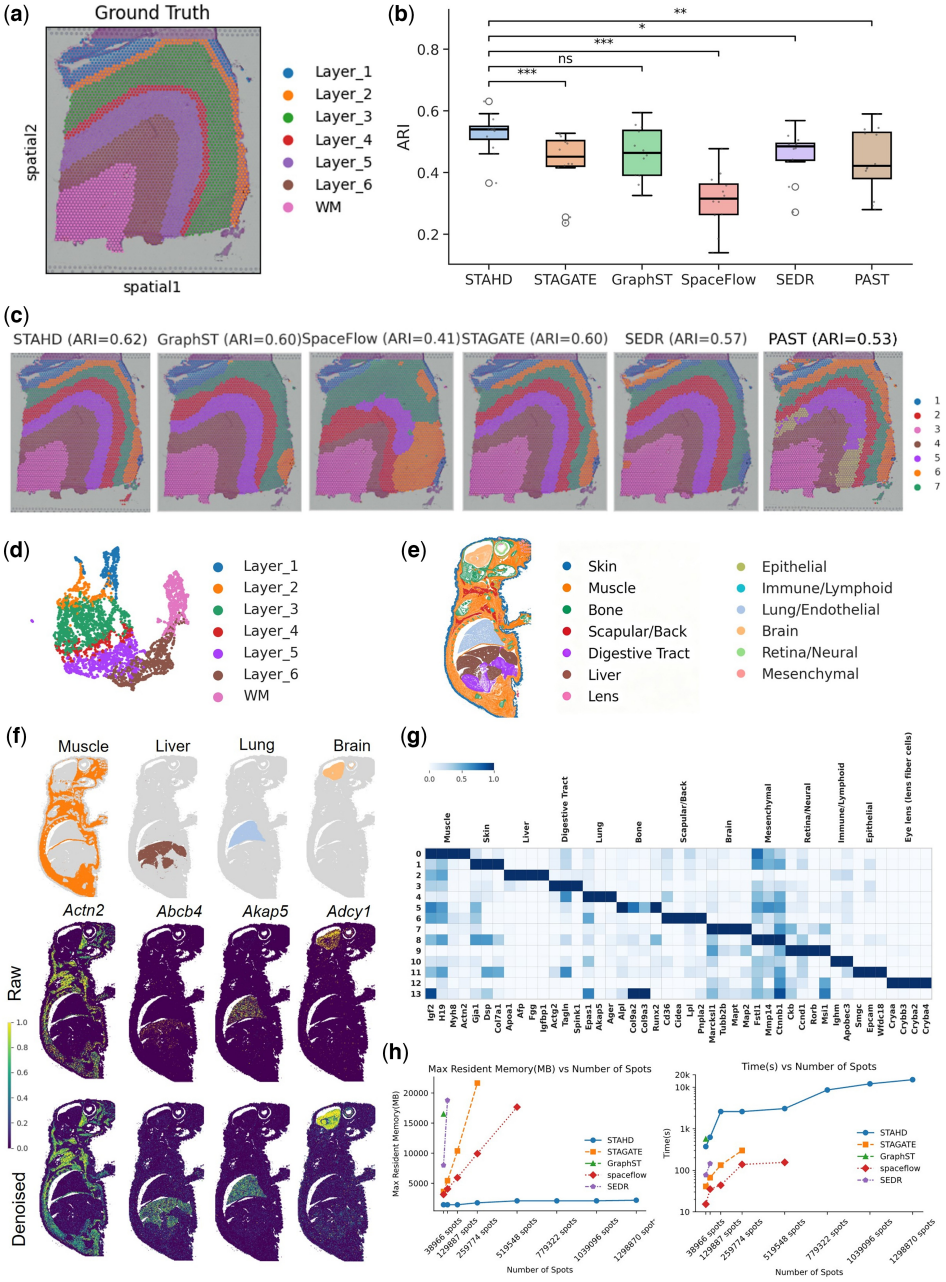
STAHD performance analysis: benchmarking on human DLPFC slices and adult mouse slice. (a) Ground-truth manual annotations of human DLPFC slices. (b) Benchmarking across 12 DLPFC slices: STAHD achieves the highest median ARI, significantly outperforming alternative methods (STAGATE, GraphST, SpaceFlow, SEDR, and PAST) based on Wilcoxon signed-rank tests. (c) Clustering results on slice 151 673: STAHD achieves the best concordance with ground-truth layers (ARI = 0.62), compared to other methods. (d) UMAP visualizations for DLPFC data section 151 673, using low-dimensional embeddings from STAHD, colored by the layer annotation of spots. (e) STAHD identifies spatial domains in the xenium_whole_adult_mouse dataset, capturing diverse tissue types such as skin, muscle, lung, and brain. (f) Spatial organization substructure of some of the marked genes and visualizations of the raw spatial expressions and STAHD denoised ones in the xenium_whole_adult_mouse. (g) Heatmap of tissue-specific marker gene expression profiles in the xenium_whole_adult_mouse dataset. (h) Scalability analysis: STAHD maintains low memory usage and training time, with memory scaling sublinearly with the number of spots, outperforming other methods.

To systematically evaluate the performance of STAHD, we benchmarked it against five widely used unsupervised spatial domain detection methods: SEDR, STAGATE, SpaceFlow, GraphST, and PAST using the 10x Visium human dorsolateral prefrontal cortex (DLPFC) dataset consisting of 12 tissue sections. Manual annotations of cortical layers (layers 1–6 and white matter) were used as the gold standard for evaluation (Fig. 2a). This demonstrates that STAHD successfully balances computational efficiency with accuracy. Further quantitative analysis based on ARI, NMI, AMI, and Homogeneity across the 12 sections (Fig. 2b) confirmed that STAHD achieved the highest median ARI (0.62). In addition, Fig. 1, available as supplementary data at *Bioinformatics* online, provides a more comprehensive comparison across multiple metrics. The bar plots (Fig. 1a, available as supplementary data at *Bioinformatics* online) show that STAHD attains the overall highest or comparable scores among all benchmark methods, while the box plots (Fig. 1b, available as supplementary data at *Bioinformatics* online) illustrate its robust performance across datasets, characterized by higher median scores and reduced variance. Outperforming all competing methods even though they used full-graph training (Fig. 2c). To assess the statistical significance of these performance differences, pairwise Wilcoxon signed-rank tests were conducted between STAHD and each benchmark method, with *P*-values adjusted using the Benjamini–Hochberg procedure and significance thresholds set at *P* < .05 (*), *P* < .01 (**), and *P* < .001 (***) (Table 5, available as supplementary data at *Bioinformatics* online). These results validate the superior overall performance of STAHD in terms of both accuracy and scalability. It is worth noting that ARI was computed only for the DLPFC dataset with available manual annotations, whereas for other datasets lacking ground-truth labels, performance was assessed through biological validation, including morphology, marker genes, and functional coherence.

Moreover, by integrating spatial coordinates, STAHD precisely computed the relative distances between spatial domains and visualized the spatial spots via UMAP dimensionality reduction (Fig. 2d). Taking sample #151673 as an example, the UMAP projection generated from STAHD embeddings clearly depicted a hierarchical spatial gradient extending from superficial Layer 1 to deeper Layer 6 and white matter, faithfully recapitulating the anatomically ordered structure of the human cerebral cortex. This observation is highly consistent with known neurobiological principles, as adjacent cortical layers exhibit functional coupling (Gilmore and Herrup 1997). We assessed the sensitivity of STAHD to subgraph size on the Xenium whole adult mouse dataset by varying the batch size parameter (512–8192). Clustering consistency across different settings was evaluated using Cramér’s V, which is robust to label permutations and does not require ground-truth annotations. As shown in Fig. 2a, available as supplementary data at *Bioinformatics* online, the results remained highly consistent (Cramér’s *V* ≈ 1), indicating that STAHD is robust to subgraph size and that varying batch size does not substantially affect spatial domain detection.

### 3.3 Spatial domain identification on the xenium whole adult mouse dataset using STAHD

STAHD was further applied to the 10x Xenium Adult Mouse Dataset, which includes 1 298 870 cells. The method identified major anatomical regions such as skin, muscle, skeletal areas, brain, and retina nerve tissues. It also delineated spatial domains enriched in epithelial cells, immune clusters, pulmonary endothelial, and mesenchymal cells (Fig. 2e).

To validate these domains, we examined region-specific marker genes. The clustering results showed strong consistency with known biological annotations. Notably, *Actn2* showed high expression in muscle tissue (Murphy and Young 2015), *Abcb4* was significantly expressed in the liver (Lipiński *et al.* 2021), *Akap5* was enriched in the lung (Lester *et al.* 1997), and *Adcy1* predominantly localized to brain regions (Wong *et al.* 1999). The denoised data by STAHD revealed clearer spatial expression for markers such as *Abcb4, Akap5*, and *Adcy1*, which were fragmented in the original dataset (Fig. 2f). This demonstrates that STAHD enhances data interpretability and preserves biological signals.

We further visualized gene expression across anatomical regions using a heatmap (Fig. 2g). Region-enriched genes included *Igf2, H19, Myh8*, and *Actn2* in muscle, and *Apoa1, Fgg*, and *Afp* in liver, confirming the biological relevance of STAHD-identified domains.

STAHD also demonstrated excellent memory efficiency. It required only 2.1 GB of GPU memory to analyze over 1 million cells. As the cell number increased from 52 000 to 130 000, runtime increased from 4000 to 20 000 s (Fig. 2h), much lower than the expected 2.5× growth. In contrast, mainstream methods failed due to out-of-memory errors. These results confirm that STAHD not only scales effectively for ultra-large spatial datasets but also maintains high analytical accuracy and stability. Although STAHD scales efficiently to large datasets, its computational efficiency is less competitive on very small datasets due to the fixed overhead of preprocessing steps such as graph coarsening and mini-batch training. For datasets with fewer than ∼50 000 spots, traditional methods (e.g. STAGATE) are expected to run faster with comparable accuracy. In contrast, STAHD is particularly advantageous for medium-to-large-scale analyses, where it enables efficient processing of hundreds of thousands to millions of spots with substantially reduced memory usage.

### 3.4 STAHD reveals fine-scale spatial domains in human lymph node CosMx data

**Figure 3.**
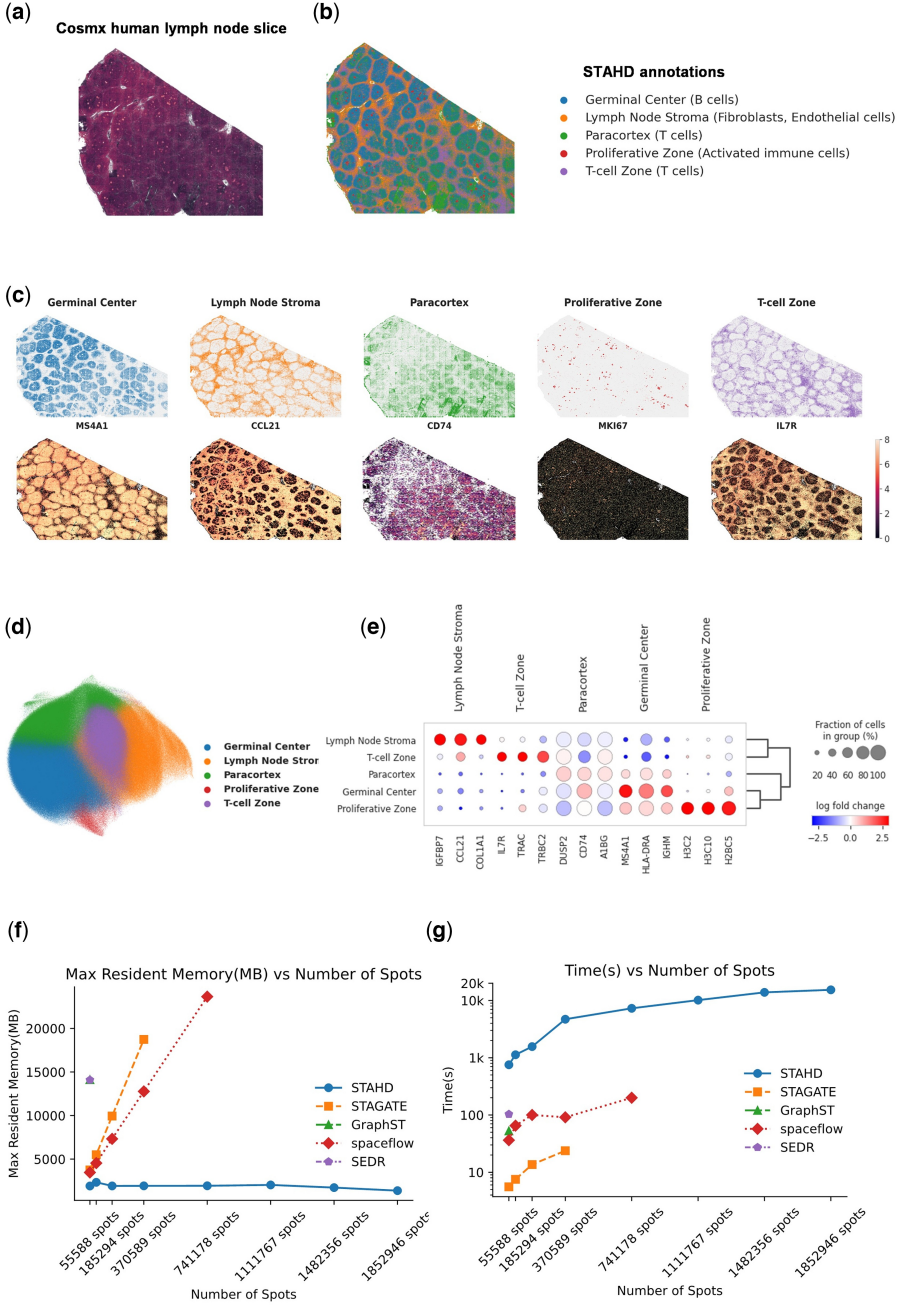
Spatial domain delineation and cellular mapping in human lymphoid tissue using STAHD. (a) High-resolution morphological image of CosMx lymph node tissue section. (b) Spatial domains identified by STAHD reveal anatomically coherent structures. (c) Mapping of Leiden clusters and representative marker gene expression in spatial context. (d) UMAP projection shows transcriptional heterogeneity across spatial domains. (e) Top three differentially expressed genes (log_2_ fold change) for each Leiden cluster. (f) Memory consumption scales linearly with number of cells in CosMx lymph data. (g) Runtime analysis of STAHD with increasing cellular resolution in CosMx lymph tissue.

We applied STAHD to the CosMx human lymph node single-cell ST dataset (CosMx Human Lymph Node FFPE Dataset 2025), which comprises 1 852 946 spots and 6520 genes, the largest available single-cell ST dataset. Figure 3a presents the high-resolution morphological image of the tissue section, providing histological reference for subsequent spatial clustering analysis.

Focusing on organ-level functional structure identification, we clustered five spatial domains. STAHD successfully delineated the germinal center, T-cell zone, activated immune cell region, stromal region, and T-cell-enriched zone (Fig. 3b), with spatial distribution patterns highly consistent with anatomical tissue structures. Further spatial mapping analysis integrating representative marker genes, such as *CCL21* (Förster *et al.* 1999), *CD74* (Serviss *et al.* 2018), *MKI67* (Scholzen and Gerdes 2000), and *IL7R* (Mazzucchelli and Durum 2007), enabled biological functional annotation of distinct spatial domains (Fig. 3c). The results showed strong concordance between each functional domain and the spatial enrichment of corresponding marker genes, validating STAHD’s accuracy in resolving organ architecture.

**Figure 4.**
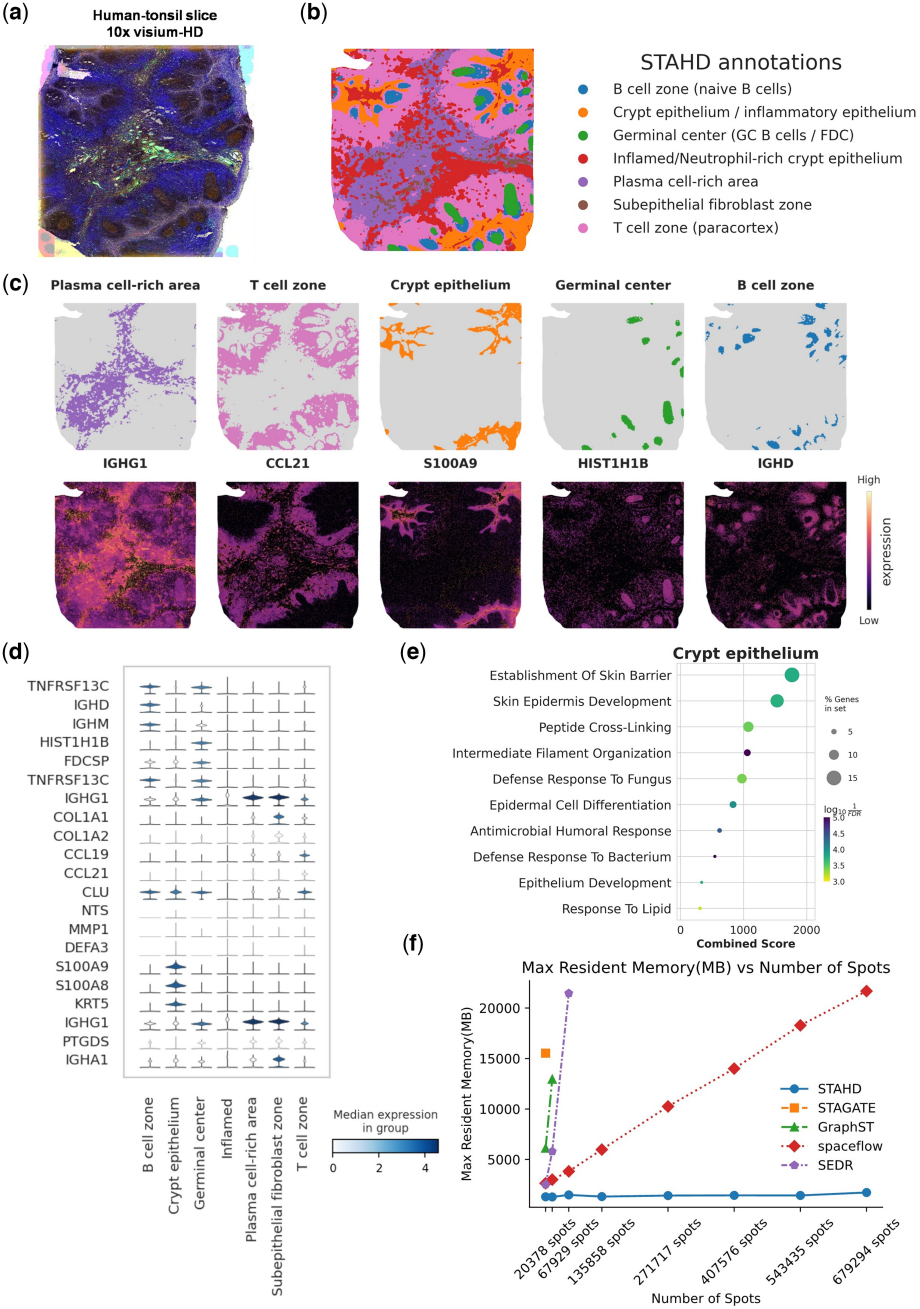
Spatial domain identification and biological annotation of human tonsil using STAHD. (a) Morphological image of human tonsil tissue slice captured by the 10x Visium-HD platform. (b) Spatial domain identification results obtained by STAHD, including B cell zone, crypt epithelium, germinal center, inflamed/neutrophil-rich crypt epithelium, plasma cell-rich area, subepithelial fibroblast zone, and T-cell zone. (c) Spatial expression heatmaps of representative marker genes, showing domain-specific expression patterns across different anatomical structures. (d) Stacked violin plots of marker gene expression levels in annotated spatial domains, illustrating the transcriptional heterogeneity within human tonsil tissue. (e) GO biological process enrichment analysis of the crypt epithelium domain. (f) Comparison of memory consumption for STAHD, STAGATE, GraphST, spaceflow, and SEDR on large-scale Xenium spatial transcriptomics datasets.

UMAP results (Fig. 3d) show that STAHD improves the separation of spatial structures, revealing clear and distinct clusters that reflect both local and global spatial features. All UMAP visualizations were generated using a uniform Scanpy workflow with fixed parameters to ensure comparability across figures. Further analysis of cluster-specific differentially expressed genes identified the top three marker genes per cluster (Fig. 3e), indicating unique and significant expression patterns across spatial regions. For example, *IGFBP7* (Broughton *et al.* 2010) and *CCL21* (Förster *et al.* 1999) were highly expressed in the lymph node stroma, *IGHM* (Seifert *et al.* 2015) were enriched in the germinal center, and *IL7R* (Mazzucchelli and Durum 2007) and *TRBC2* (Emerson *et al.* 2017) were predominantly expressed in the T-cell zone, confirming the biological relevance of STAHD’s spatial clustering results. Functional enrichment analysis (Fig. 4b, available as supplementary data at *Bioinformatics* online) further revealed significant differences in immune-related pathways among spatial domains. The germinal center was predominantly enriched for B cell receptor signaling and B cell activation pathways, whereas the T-cell zone was enriched in T-cell activation and T-cell receptor signaling pathways, reflecting active immune microenvironments in distinct regions and underscoring STAHD’s utility in dissecting spatial microenvironmental heterogeneity.

In terms of computational performance, STAHD demonstrated superior efficiency compared to STAGATE, GraphST, SpaceFlow, and SEDR (Fig. 3f and g). For datasets exceeding 10 000 cells, STAHD maintained an O(*n*log *n*) time complexity, while comparative methods exhibited quadratic O(*n*^2^) growth. Thanks to its memory-optimized design, STAHD stably completed analyses of million-cell scale single-cell ST data, avoiding common out-of-memory issues encountered by mainstream methods, and demonstrating excellent scalability and computational robustness.

### 3.5 Spatial structure identification and functional annotation of human tonsil based on STAHD

We applied the STAHD method to analyze human tonsil ST data generated by the 10x Visium-HD platform. The morphological image of the tissue section (Fig. 4a) clearly displays the typical structural features, providing an intuitive spatial context for subsequent spatial domain segmentation and marker gene localization. We then performed spatial clustering of cellular populations within the tissue section Based on differential gene expression analysis and spatial expression patterns, we biologically annotated the resulting clusters, delineating distinct functional spatial domains and their potential biological roles within the tissue.

STAHD partitioned the tonsil tissue into seven biologically meaningful spatial microenvironments (Fig. 4b), including naive B cell zones, crypt epithelium, germinal centers (GC B cells/FDC), inflamed/neutrophil-enriched crypt epithelium, plasma cell-enriched zones, submucosal fibroblast regions, and T-cell zones (paracortex). This spatial distribution pattern faithfully reflects the typical immune microenvironment architecture of tonsil tissue, with distinct immune cell populations exhibiting clear spatial partitioning and heterogeneity, highlighting the organ’s role as a key mucosa-associated lymphoid tissue with important immunoregulatory functions.

To further validate the accuracy of spatial domain annotations, representative marker genes were selected for spatial expression visualization (Fig. 4c). The results show that *IGHG1* (Chen *et al.* 2023) is primarily expressed in the plasma cell-enriched region, *CCL21* (Förster *et al.* 1999) is enriched in the T-cell zone, *HIST1H1B* (Ripoll *et al.* 2008) is highly expressed in germinal centers, and *IGHD* (Seifert *et al.* 2015) is mainly localized to the B cell zone. These spatially restricted expression patterns are highly consistent with histological structures, confirming the biological validity and accuracy of the STAHD-based spatial domain delineation.

Stacked violin plots (Fig. 4d) showed distinct marker gene expression across spatial domains, such as *IGHG1* and *IGHM* in plasma cell regions, *CCL21* in the T-cell zone, and *HIST1H1B* in germinal centers, highlighting transcriptional heterogeneity. GO analysis of the crypt epithelium (Fig. 4e) revealed enrichment in epithelial barrier and immune response processes, supporting its functional annotation.

Finally, we compared memory consumption of STAHD with other mainstream spatial omics analysis methods, including STAGATE, GraphST, SpaceFlow, and SEDR. When applied to large-scale Xenium ST data (Fig. 4f). STAHD demonstrated consistently lower memory usage while analyzing large-scale cellular data, significantly outperforming other methods.

### 3.6 STAHD reveals complex and refined spatial structures in human breast cancer tissue through spatial clustering analysis

**Figure 5.**
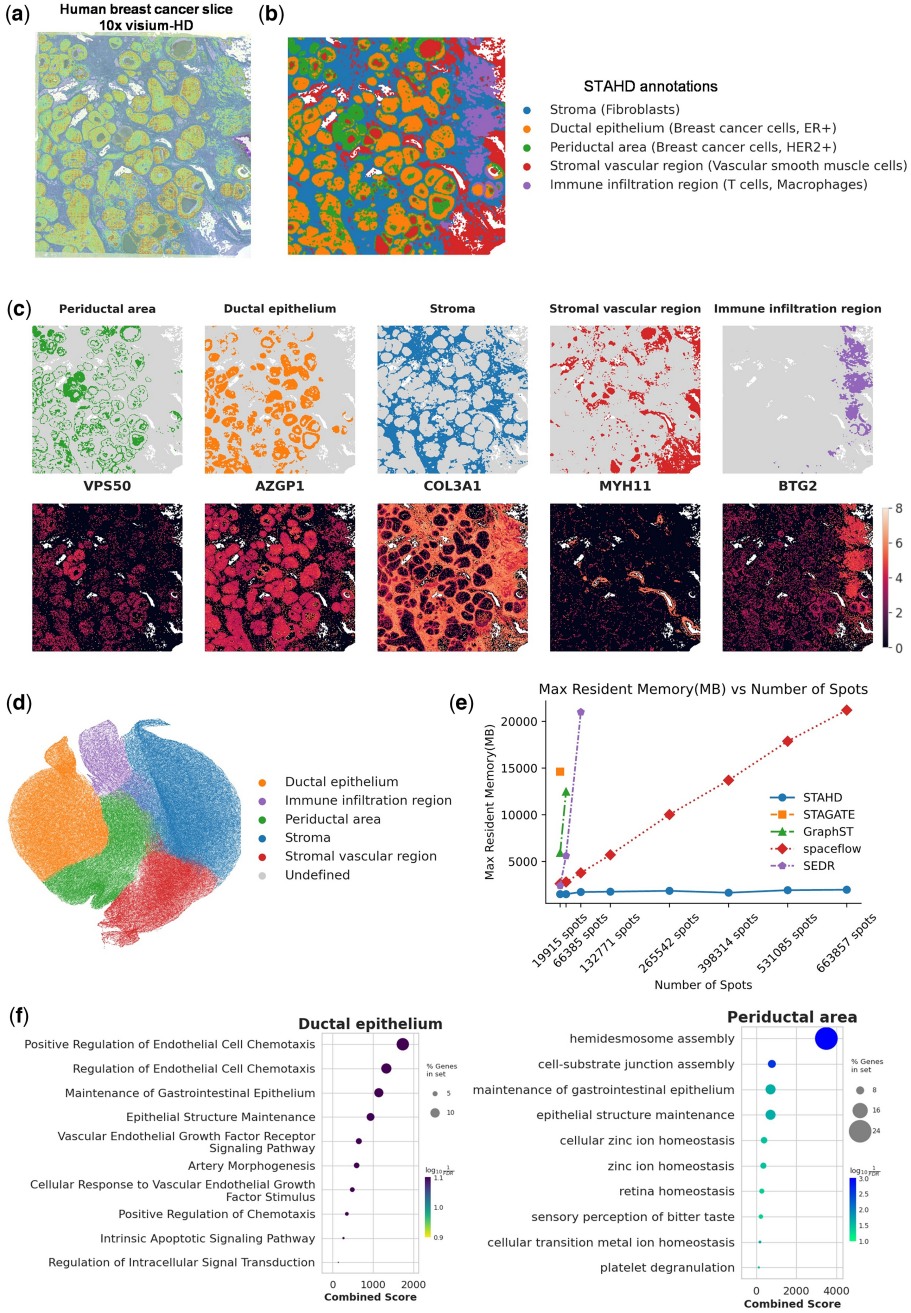
Spatial mapping of tissue structures and transcriptional heterogeneity in human breast cancer using STAHD. (a) High-resolution spatial transcriptomic map of a human breast cancer section, showing total UMI counts per bin to reveal spatial variation in transcriptional activity. (b) Spatial domains identified within the tissue, corresponding to distinct histological regions. (c) Distribution of Leiden clusters and key marker genes, highlighting cell type-specific spatial patterns. (d) UMAP visualization of CosMx lymphatic cells, showing transcriptional heterogeneity among immune populations. (e) Memory usage as a function of cell number for the high-resolution breast cancer dataset. (f) GO enrichment analysis of stromal vascular regions, indicating enrichment in immune-related and metabolic processes.

We applied STAHD approach to analyze human breast cancer ST data generated using the 10x Visium-HD platform, systematically exploring spatial heterogeneity within the tumor microenvironment. Breast cancer exhibits pronounced intratumoral heterogeneity, characterized by complex cellular compositions and spatial structural features (Shan *et al.* 2022, Bai *et al.* 2024). Figure 5a illustrates the spatial transcriptomic map of the breast cancer tissue section, with total UMI counts reflecting spatial variation in transcriptional activity and serving as the basis for spatial clustering.

We finely delineated spatial regions within the tumor tissue section (Fig. 5b). The identified domains include the stroma (rich in fibroblasts), ductal epithelium (ER+ breast cancer cells), periductal area (HER2+ breast cancer cells), stromal vascular region (vascular smooth muscle cells), and immune infiltration region (T-cells and macrophages). The stroma primarily localizes to interstitial spaces and is fibroblast-rich; the ductal epithelium consists of clustered ER+ cancer cells surrounding glandular structures; the periductal area is dominated by HER2+ cancer cells located adjacent to ducts; the stromal vascular region is enriched in vascular smooth muscle cells near blood vessels; and the immune infiltration region mainly comprises T-cells and macrophages, frequently found at tumor margins and areas of necrosis. This spatial distribution reveals the heterogeneity and spatial organization of distinct cellular populations within breast cancer tissue, providing a foundation for dissecting tumor microenvironment cellular interactions and spatial dependency mechanisms.

We further performed differential expression analysis (Fig. 5c), selecting representative marker genes for each spatial domain and visualizing their expression patterns. *COL1A1* (Oskarsson 2013) is highly expressed in the periductal area; *SLC39A6* (Taylor *et al.* 2008) predominantly localizes to the ductal epithelium; *CD24* (Kristiansen *et al.* 2003) shows widespread expression in the stroma; *MYH11* (Hao *et al.* 2021) integrated analysis of multimodal is specifically enriched in the stromal vascular region; and *TCIM* exhibits elevated expression within the immune infiltration region. The spatial expression profiles of these markers corroborate the STAHD-derived spatial annotations, further validating the accuracy of domain identification while unveiling molecular characteristics and spatial distributions of different cellular populations within the tumor tissue.

UMAP visualization (Fig. 5d) shows that cells form distinct clusters corresponding to spatial domains, confirming alignment between spatial location and transcriptomic profiles. STAHD achieves lower memory usage than SEDR, SpaceFlow, and GraphST across various cell counts (Fig. 5e), demonstrating superior scalability. GO enrichment analysis (Fig. 5f) reveals that ductal epithelium is involved in angiogenesis and epithelial maintenance, while the periductal area is enriched in processes related to adhesion and metal ion regulation, supporting the functional relevance of spatial domains.

## 4 Discussion

STAHD effectively mitigates high memory consumption challenges faced by traditional full-graph-based methods when analyzing ultra-large-scale ST data. On million-cell datasets generated by the Xenium platform, STAHD completes training within 4 h on a 24 GB GPU, which cannot be accomplished by mainstream methods such as STAGATE and GraphST. In contrast to other approaches, STAHD avoids holding the entire spatial neighbor graph in memory. By partitioning the graph into compact subgraphs and training them in mini-batches, STAHD prevents memory overload while preserving local structures. This strategy enables stable analysis of million-cell datasets with only 2.1 GB of GPU memory, highlighting its scalability advantage. The graph coarsening step in STAHD conceptually resembles “metacells” in single-cell analysis, as both aggregate similar units to reduce complexity. However, unlike metacell methods that group cells by molecular similarity, STAHD’s coarsening is driven by spatial adjacency and graph topology, and the coarsened units are later refined back to the original graph. These supernodes could nevertheless be explored as spatial metacells for downstream analysis in future work. At present, STAHD primarily builds adjacency graphs based on spatial proximity, without fully incorporating multimodal spatial omics data—such as histological image features, proteomics (Cheng *et al.* 2019), and spatial metabolomics. Expanding its integration of these modalities is a key direction for future work to enhance its ability to capture complex cross-hierarchical spatial structures. We further examined the impact of different clustering algorithms on spatial domain identification. The results demonstrated that STAHD produced generally consistent spatial structures across Leiden, *K*-means, and Mclust clustering, with only minor local variations (Fig. 3, available as supplementary data at *Bioinformatics* online).

In our experiments, the number of clusters was determined by dataset characteristics: seven for DLPFC (matching ground-truth annotations), and empirically chosen for others to ensure stability and biological interpretability, resulting in 13 for the Xenium mouse dataset, five for human lymph node (CosMx), seven for tonsil, and five for breast cancer tissue. From a practical perspective, because STAHD introduces additional preprocessing overhead, it is particularly suitable for medium-to-large ST datasets (hundreds of thousands to millions of spots) or in scenarios where memory constraints make full-graph training infeasible; for smaller datasets (on the order of 10^4^ spots or fewer), existing full-graph methods may provide faster turnaround. Detailed runtime and memory usage measurements for all datasets are provided in Tables 1–4, available as supplementary data at *Bioinformatics* online, alongside the performance plots, to facilitate a direct comparison with other methods. Furthermore, as multiomics play an important role in deciphering tissues (Jin *et al.* 2024), we will extend our method to spatial multiomics in the near future (Huang *et al.* 2025).

## Supplementary Material

btaf619_Supplementary_Data

## Data Availability

The source code of STAHD used in this study has been archived on Zenodo (https://doi.org/10.5281/zenodo.17214602), corresponding to version v1.0.0 that was used to generate the results presented in this article. The latest development version is available on GitHub at https://github.com/Little-Eel/STAHD. All spatial transcriptomics datasets used in this study are publicly available. Detailed sources and download links are listed below: 10x Visium human dorsolateral prefrontal cortex (DLPFC) dataset and tutorials: https://support.10xgenomics.com/spatial-gene-expression/datasets/1.2.0/V1_Human_DLPFC. Xenium platform whole adult mouse brain dataset (xenium_whole_adult_mouse), including data and tutorials: https://www.10xgenomics.com/datasets/xenium-prime-ffpe-neonatal-mouse. CosMx SMI human lymph node dataset (Cosmx lymph) from NanoString: https://nanostring.com/products/cosmx-spatial-molecular-imager/ffpe-dataset/cosmx-human-lymph-node-ffpe-dataset/. 10x Genomics Visium-HD human breast cancer dataset (FFPE-IF): https://www.10xgenomics.com/datasets/visium-hd-cytassist-gene-expression-libraries-human-breast-cancer-ffpe-if. 10x Genomics Visium-HD human tonsil dataset (fresh frozen, IF): https://www.10xgenomics.com/datasets/visium-hd-cytassist-gene-expression-human-tonsil-fresh-frozen-if.
