# Supplementary material for: STAHD: a scalable and accurate method to detect spatial domains in high-resolution spatial transcriptomics data
Source: Bioinformatics. 2025 Nov 10;42(1):btaf619. doi: 10.1093/bioinformatics/btaf619 (PMC12790823; doi:10.1093/bioinformatics/btaf619)
Supplement: btaf619_Supplementary_Data [file btaf619_supplementary_data.doc]

# Supplementary Information

# STAHD: a scalable and accurate method to detect spatial domains in high-resolution spatial transcriptomics data

Zhihua Du^1^, Di Wang^1,2^, Qiyi Chen^1^, Yuehua Ou^1^, Xinlei Huang^2^, Xiang Zhou^3,*^, and Xubin Zheng^2,*^

^1^College of Computer Science and Software Engineering, ShenZhen University, Shenzhen, China,

^2^Guangdong Provincial Key Laboratory of Mathematical and Neural Dynamical Systems, School of Computing and Information Technology, Great Bay University, Guangdong, China.

^3^Guangdong Institute of Intelligence Science and Technology, Hengqin, Zhuhai, Guangdong, China.

Xubin Zheng:[xbzheng@gbu.edu.cn](mailto:xbzheng@gbu.edu.cn);

## 1 Supplementary Notes

## Datasets

### Human dorsolateral prefrontal cortex (DLPFC) dataset

This dataset was generated using the 10x Genomics Visium platform and comprises 12 brain tissue sections from 3 adult donors. Each section contains between 3,498 and 4,789 spatial capture spots, covering six neocortical layers (Layers 1–6) and the white matter region. Manually curated anatomical annotations based on histological morphology were provided in the original study, serving as ground-truth labels for spatial clustering evaluation (Maynar*d et a*l., 2021). This dataset was primarily used to assess the spatial domain identification accuracy of STAHD in highly structured brain tissue.

### Adult mouse Xenium whole-body dataset

This dataset was obtained using the 10x Genomics Xenium platform and contains spatial gene expression profiles of 1,298,870 cells across 5,000 genes, covering multiple anatomical regions including adult mouse skin, muscle, brain, lung, and other tissues. As one of the largest publicly available single-cell spatial transcriptomics datasets to date, it was primarily used to evaluate the scalability, memory efficiency, and spatial structure detection performance of STAHD on million-scale single-cell data.

### Human lymph node CosMx dataset

This dataset was generated using the Nanostring CosMx Spatial Molecular Imager (SMI) platform (CosMx Human Lymph Node FFPE Dataset, 2025), comprising 1,852,946 spatial capture spots and expression profiles of 6,175 genes, along with high-resolution histological images. The original dataset provides reference annotations for 18 cell subpopulations, which were used as the basis for spatial functional region delineation. This dataset was primarily employed to assess the spatial resolution and functional annotation accuracy of STAHD within complex immune microenvironments (Cabl*e et a*l., 2022a).

### Human breast cancer Visium-HD dataset

This dataset was acquired using the 10x Genomics Visium-HD platform, containing 663,857 spatial capture spots from human breast cancer tissue. It retains the spatial heterogeneity within the tumor and the spatial interactions between tumor and immune cells, making it suitable for evaluating the ability of STAHD to resolve spatial heterogeneity in the tumor microenvironment.

### Human tonsil Visium-HD dataset

Generated with the 10x Genomics Visium-HD platform, this dataset consists of 679,294 spatial capture spots from human tonsil tissue. It encompasses typical lymphoid tissue structures, including B cell zones, germinal centers, T cell areas, crypt epithelium, and plasma cell–rich regions. Spatial domain annotations were derived by integrating spatial gene expression patterns and functional enrichment analyses, enabling validation of the applicability of STAHD in lymphoid tissues.

## Data preprocessing

STAHD takes the gene expression matrix and spatial coordinates from each spatial transcriptomics (ST) tissue section as input. During data preprocessing, gene names were first standardized by removing duplicate entries to ensure the uniqueness of each gene. For datasets with ground-truth annotations (e.g., DLPFC), the corresponding annotation files were automatically loaded, and any unannotated regions were uniformly assigned to an "unknown" category to preserve the completeness of spatial annotations. For feature selection, a mean–variance relationship-based strategy was applied to high-dimensional datasets containing more than 5,000 genes. The top 3,000–5,000 highly variable genes (HVGs) were selected based on the standardized residuals of gene variance after fitting the mean–variance trend, to effectively capture the spatial heterogeneity of tissue regions. To minimize technical biases, a rigorous data preprocessing procedure was implemented. Raw counts were first normalized to a total of 10,000 per capture spot, followed by a log1p transformation to stabilize variance and improve the distribution symmetry. Finally, Z-score normalization (zero mean and unit variance) was applied along the gene dimension of the expression matrix, ensuring the comparability of expression values across genes and providing standardized input for subsequent deep learning model training.

## 2 Supplementary Figures

a

b


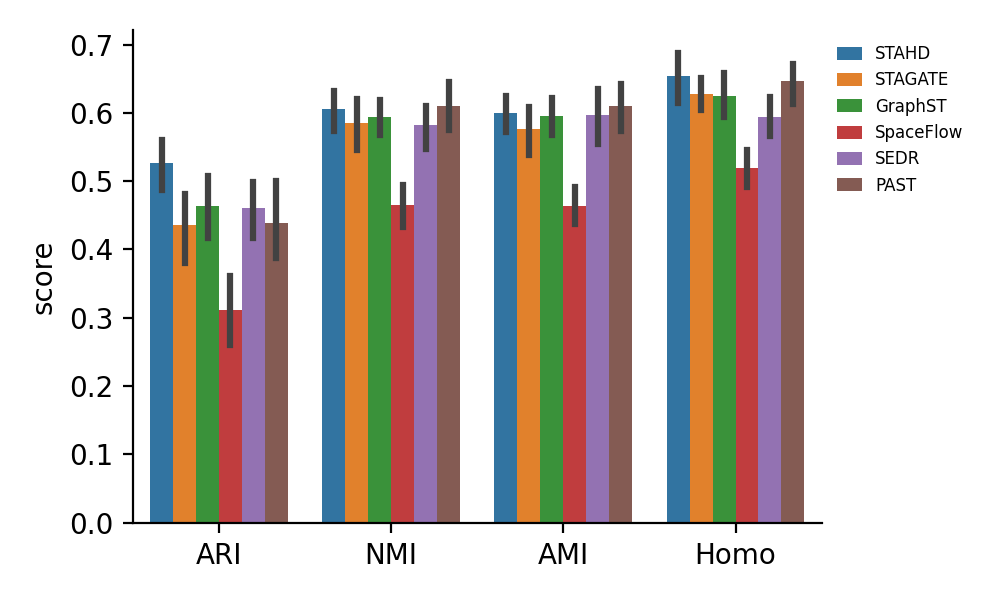

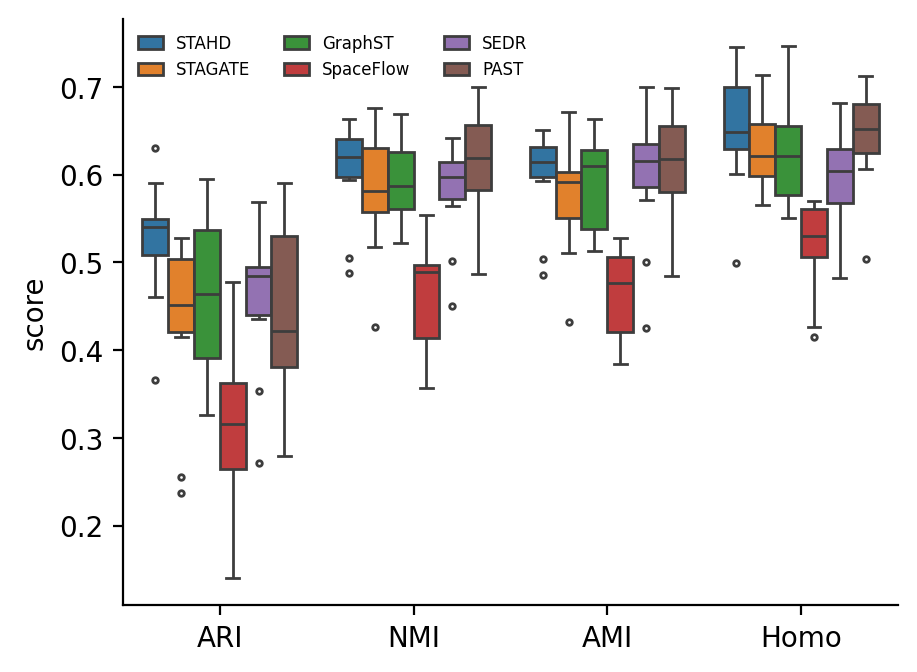

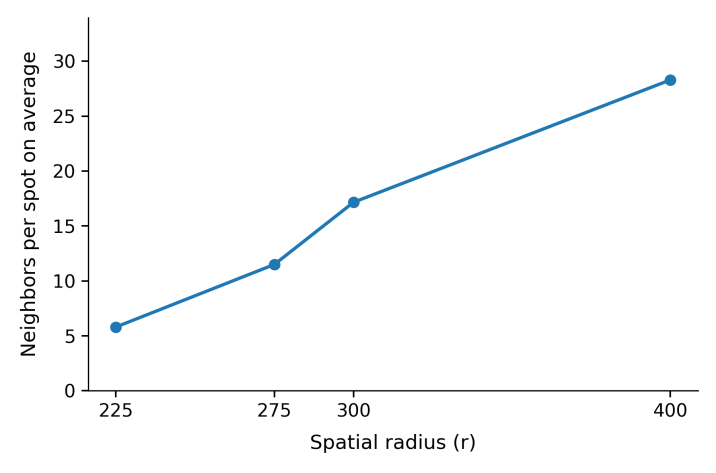

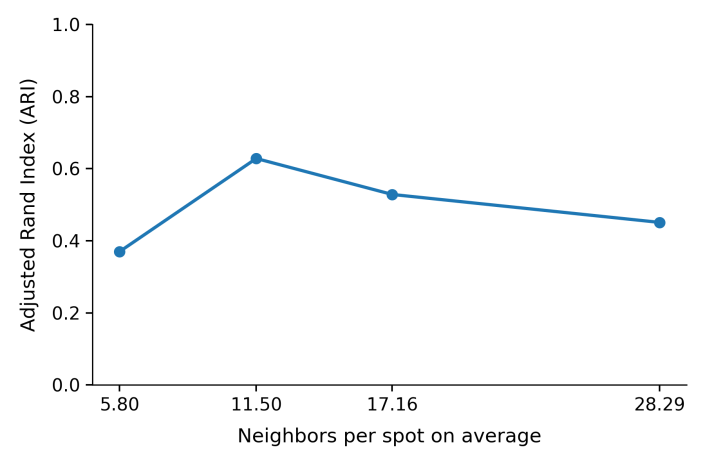


c

d

**Figure S1| Comprehensive evaluation of clustering performance and spatial sensitivity of STAHD.**

**a.** Bar plots of five methods (STAHD, STAGATE, GraphST, SpaceFlow, and SEDR) evaluated by ARI, NMI, AMI, and Homo. STAHD achieves the highest or comparable scores across all metrics.

**b.** Box plots illustrating the distribution of ARI, NMI, AMI, and Homo scores across datasets for the five methods. STAHD shows consistently robust performance with higher median scores and reduced variance.

**c.** Sensitivity analysis of the spatial radius threshold (r) on the DLPFC dataset. The plot shows the mean Adjusted Rand Index (ARI) of STAHD on the DLPFC dataset under different average neighbor counts. The performance peaks when each spot has approximately 10–30 neighbors, indicating that this range balances local spatial context and noise suppression.


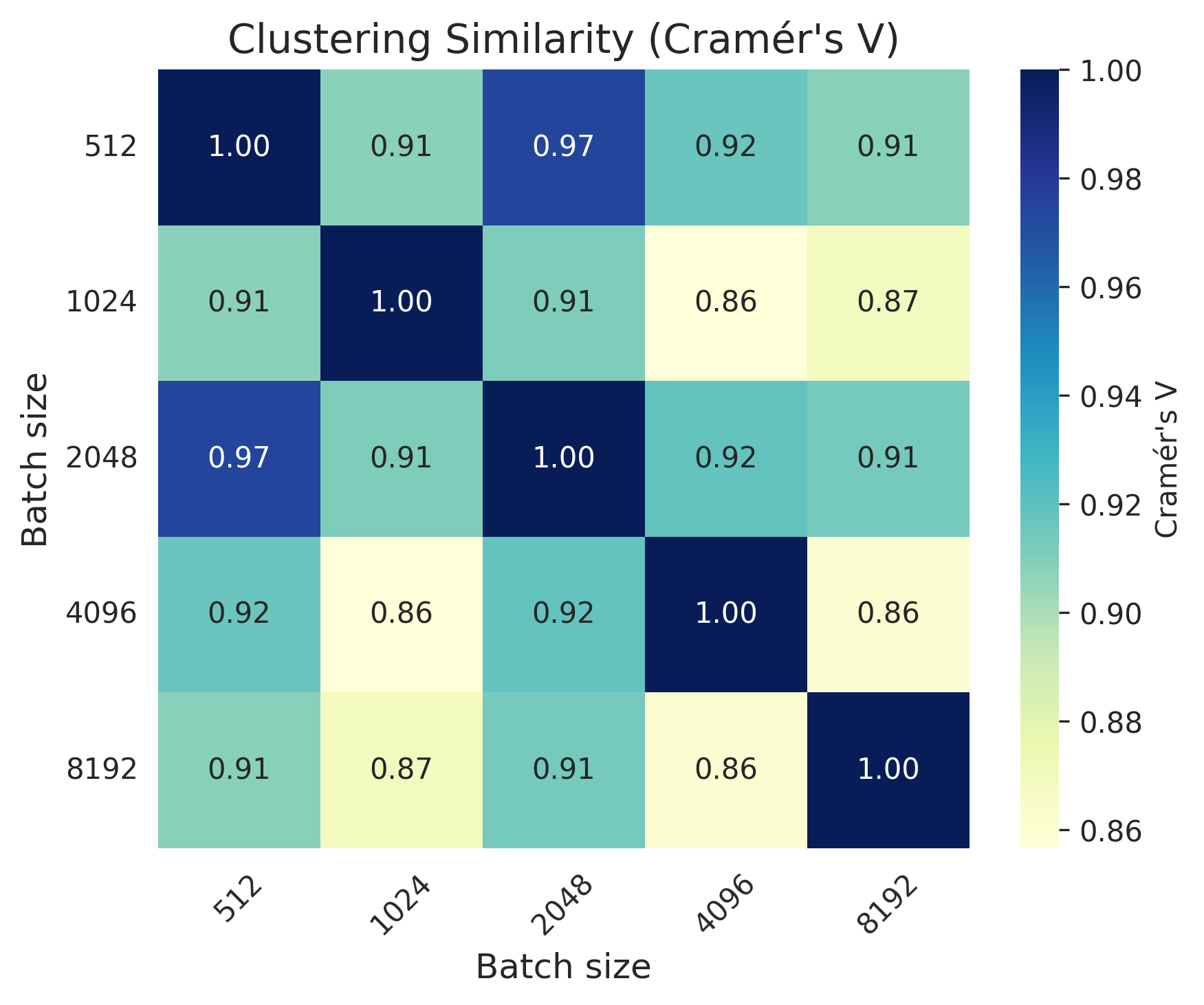
**d**. Relationship between spatial radius (r) and the average number of neighbors on the DLPFC dataset.Larger r values yield more neighbors per spot, indicating denser spatial connectivity.

a

**Figure S2| Sensitivity of STAHD to subgraph size on the Xenium whole adult mouse dataset.**

1. Each row and column corresponds to a batch size (512, 1024, 2048, 4096, 8192). The heatmap entries show the pairwise similarity of clustering results between two batch sizes. A value of 1 indicates identical clustering partitions, while values close to 0 indicate little similarity. The consistently high values (>0.9) suggest that the clustering outcomes of STAHD remain highly stable across different subgraph sizes.


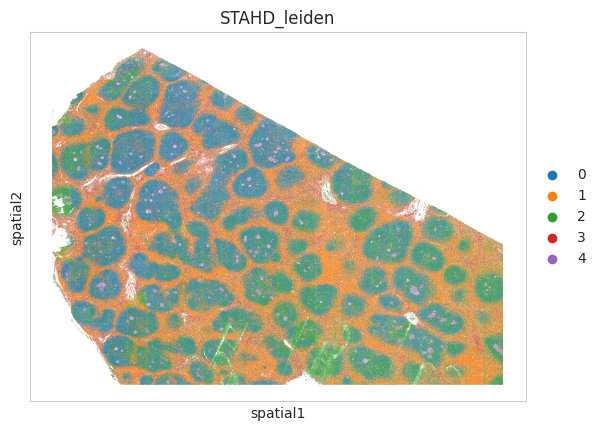


CosMx Human Lymph Node

xenium Whole Adult Mouse


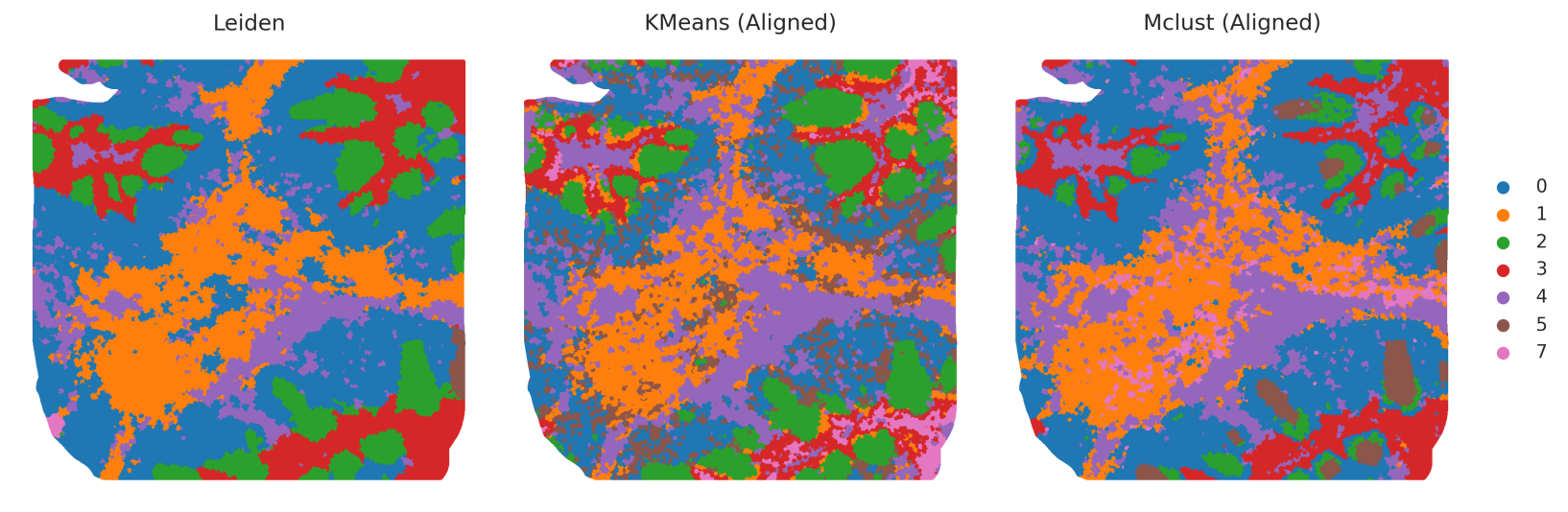


Human Tonsil


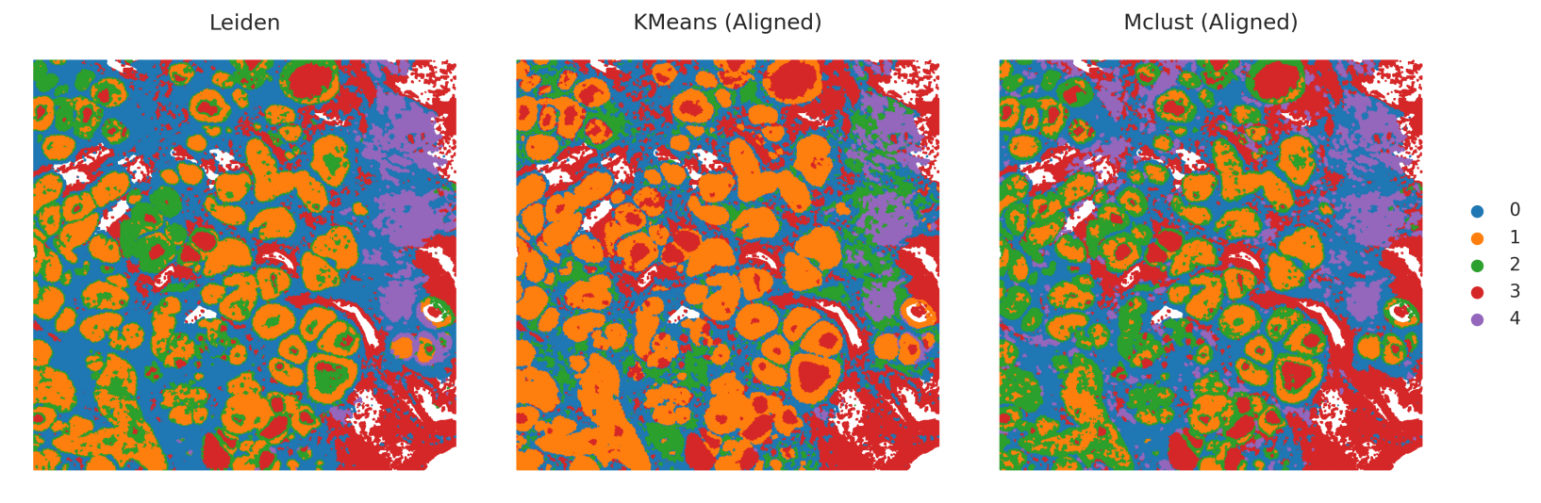


Human Breast Cancer


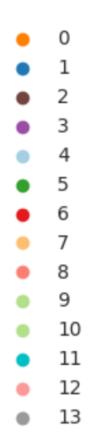

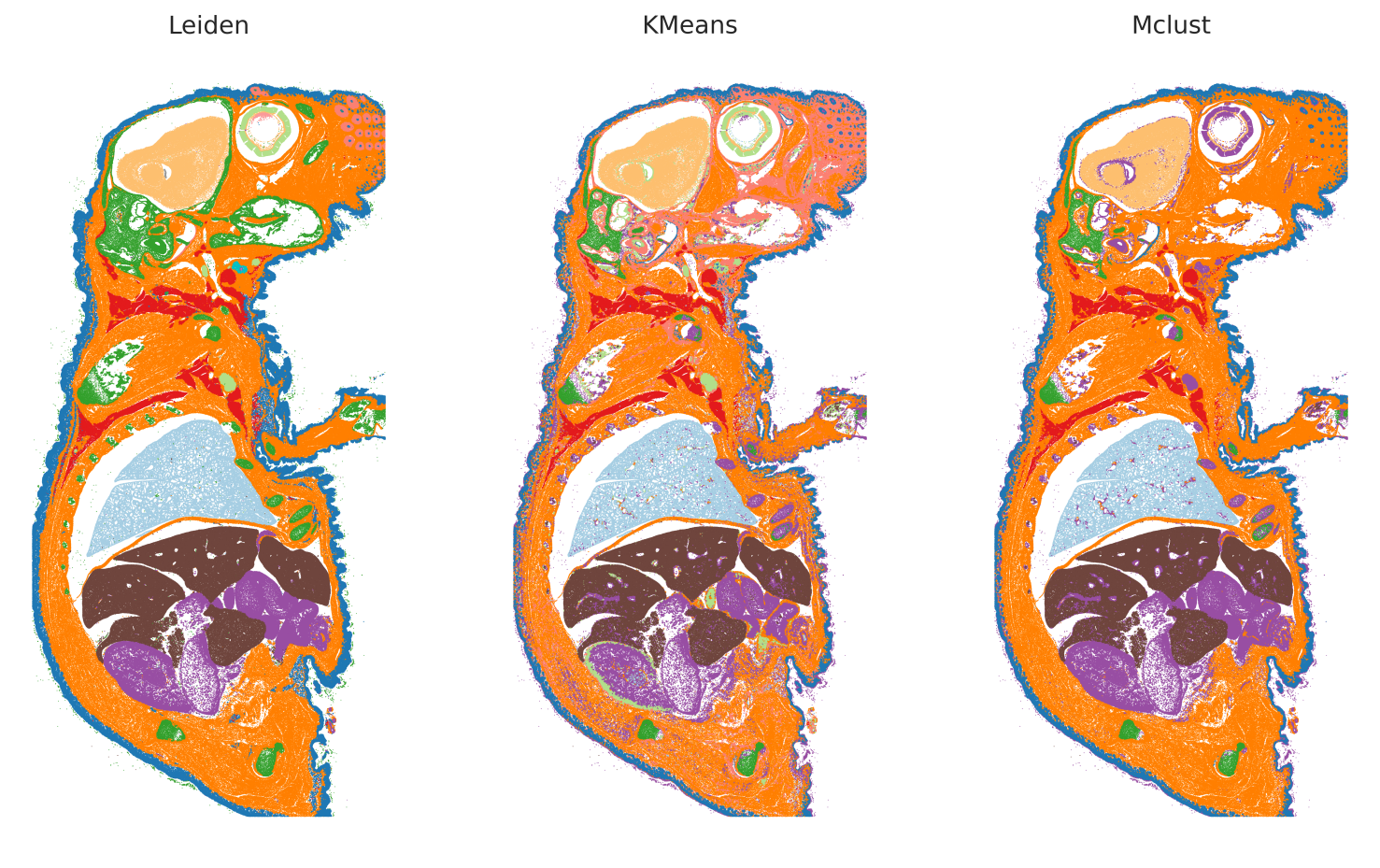

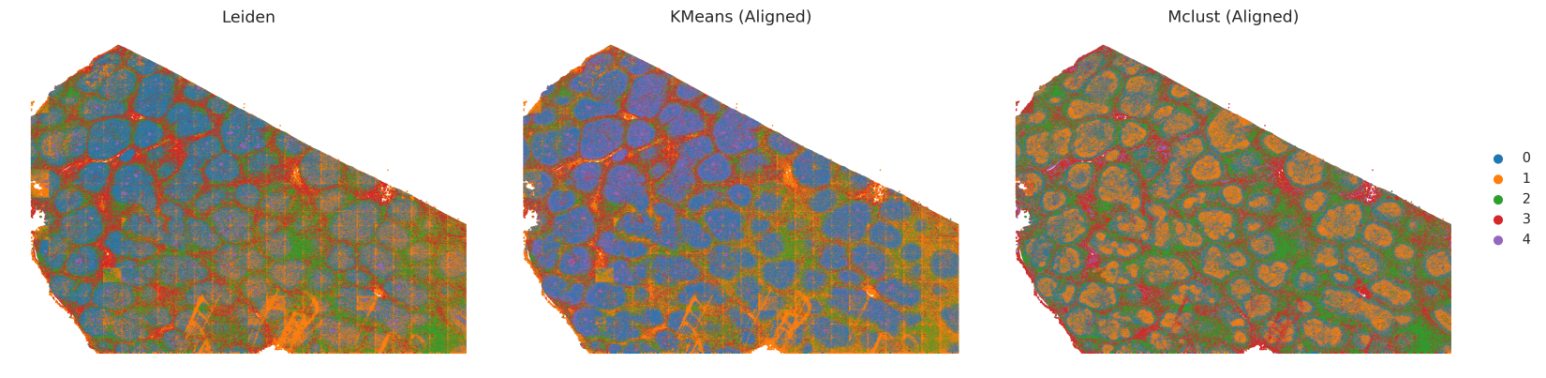

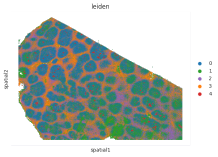

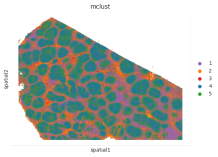


a

b

c

d

**Figure S3|. Sensitivity analysis of STAHD to the choice of clustering algorithm across multiple spatial transcriptomics datasets.**

Clustering was performed on the learned embeddings using three representative clustering methods: Leiden, K-means, and Mclust. Overall, the spatial domain detection results were largely consistent across methods, demonstrating the robustness of STAHD embeddings. However, local differences were observed in some datasets (e.g., partial merging or separation of liver and retina regions in the mouse dataset), reflecting algorithm-specific sensitivity to subtle spatial boundaries.

**a.** Xenium whole adult mouse dataset. **b.** Human lymph node (CosMx) dataset. **c.** Human tonsil (Visium-HD) dataset. **d.** Human breast cancer tissue (Visium-HD) dataset.


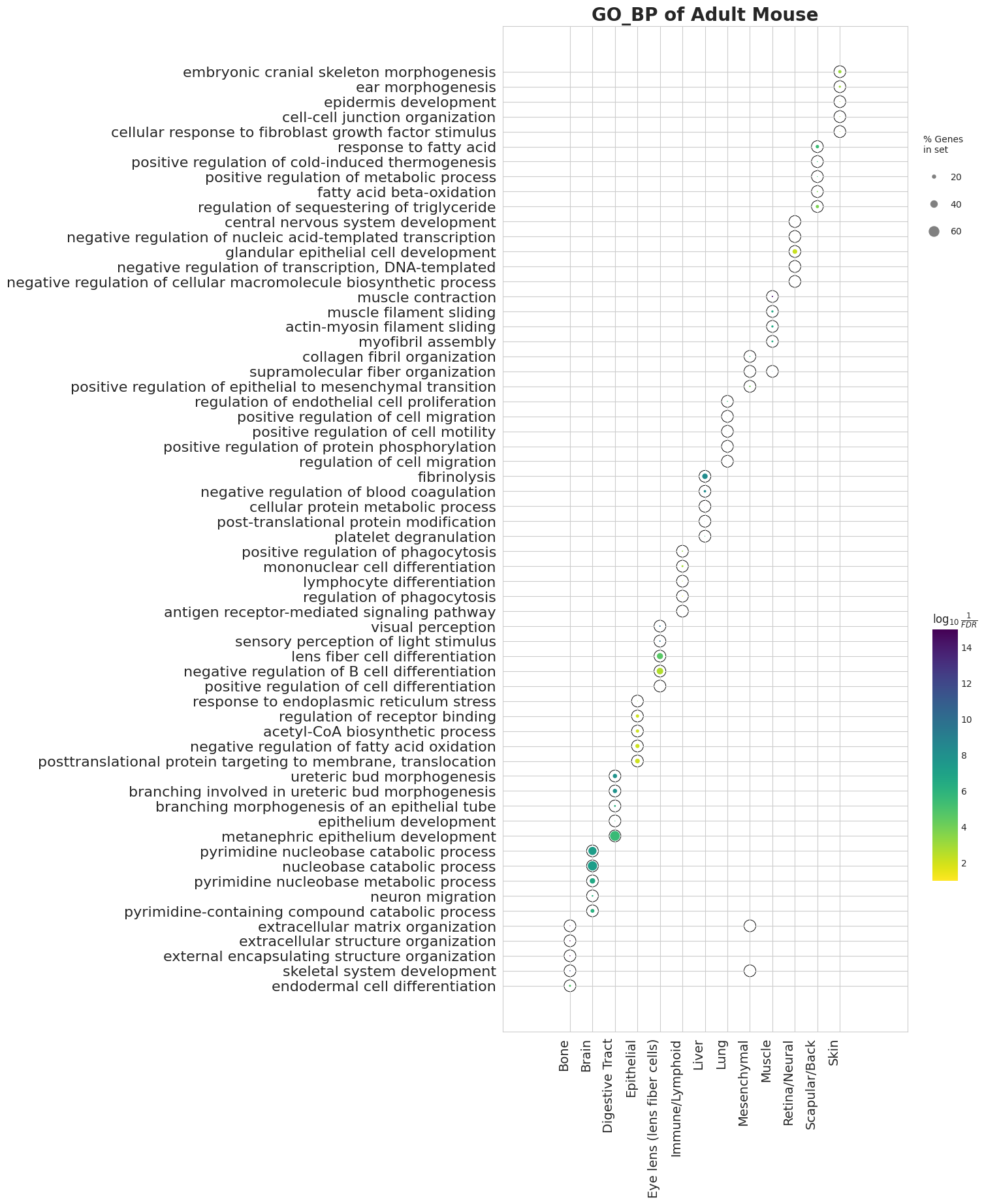


a

b


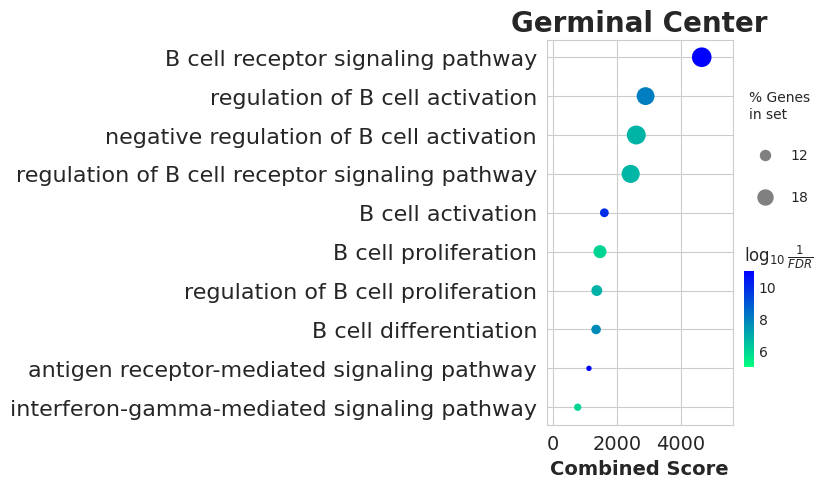

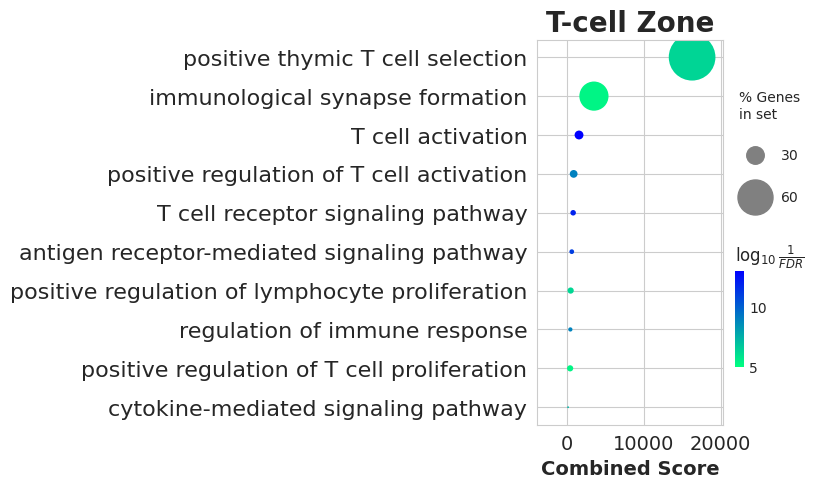


**Figure S4| GO Biological Process enrichment analysis in adult mouse and human lymphoid tissue .**

The dotplot visualizes the top enriched biological processes for each region, with dot size indicating the percentage of genes involved and color representing the significance level.

**a,**Functional Enrichment of Differentially Expressed Genes Across Adult mouse spatial domain

**b,**GO Biological Process Enrichment Analysis of Germinal Center and T-cell Zone in Human Lymph Node

**3 Supplementary Tables**

| **Dataset** | **Whole Adult Mouse（1,298,870 Spots）** | | | | | | | | | | | | | | | |
| --- | --- | --- | --- | --- | --- | --- | --- | --- | --- | --- | --- | --- | --- | --- | --- | --- |
| Method | Runtime（s） | | | | | | | | Memoryusage（GB） | | | | | | | |
|  | 3% | 5% | 10% | 20% | 40% | 60% | 80% | 100% | 3% | 5% | 10% | 20% | 40% | 60% | 80% | 100% |
| STAHD  (batch_size=256) | 372.83 | 624.31 | 2594.84 | 2594.84 | 3063.04 | 8599.25 | 12097.18 | 15204.60 | 1428 | 1428 | 1428 | 1750 | 2078 | 2078 | 2078 | 2180 |
| STAGATE | 40.94 | 66.69 | 133.13 | 300.21 | – | – | – | – | 3626 | 5428 | 10376 | 21654 | – | – | – | – |
| GraphST | 568.74 | – | – | – | – | – | – | – | 16508 | – | – | – | – | – | – | – |
| SpaceFlow | 15.28 | 35.17 | 43.76 | 138.38 | 155.09 | – | – | – | 3146 | 4062 | 5924 | 9922 | 17652 | – | – | – |
| SEDR | 76.99 | 144.95 | – | – | – | – | – | – | 7988 | 18789 | – | – | – | – | – | – |

**Table S1. Runtime and memory usage of STAHD and baseline methods on the Whole Adult Mouse dataset.**

| **Dataset** | **Human Lymph Node（1,852,946 Spots）** | | | | | | | | | | | | | | | |
| --- | --- | --- | --- | --- | --- | --- | --- | --- | --- | --- | --- | --- | --- | --- | --- | --- |
| Method | Runtime（s） | | | | | | | | Memoryusage（GB） | | | | | | | |
|  | 3% | 5% | 10% | 20% | 40% | 60% | 80% | 100% | 3% | 5% | 10% | 20% | 40% | 60% | 80% | 100% |
| STAHD  (batch_size=256) | 754.47 | 1128.78 | 1567.05 | 4710.87 | 7304.18 | 10152.23 | 13879.14 | 15362.81 | 1920 | 2344 | 1928 | 1936 | 1944 | 2040 | 1934 | 2020 |
| STAGATE | 5.57 | 7.54 | 13.65 | 23.81 | – | – | – | – | 3786 | 5508 | 9942 | 18740 | – | – | – | – |
| GraphST | 52.68 | – | – | – | – | – | – | – | 14120 | – | – | – | – | – | – | – |
| SpaceFlow | 36.34 | 65.17 | 100.21 | 91.22 | 200.19 | – | – | – | 3472 | 4546 | 7330 | 12776 | 23652 | – | – | – |
| SEDR | 103.47 | – | – | – | – | – | – | – | 14090 | – | – | – | – | – | – | – |

**Table S2. Runtime and memory usage of STAHD and baseline methods on the Human Lymph Node dataset.**

| **Dataset** | **Human Tonsil（679,294 Spots）** | | | | | | | | | | | | | | | |
| --- | --- | --- | --- | --- | --- | --- | --- | --- | --- | --- | --- | --- | --- | --- | --- | --- |
| Method | Runtime（s） | | | | | | | | Memoryusage（GB） | | | | | | | |
|  | 3% | 5% | 10% | 20% | 40% | 60% | 80% | 100% | 3% | 5% | 10% | 20% | 40% | 60% | 80% | 100% |
| STAHD  (batch_size=256) | 258.17 | 437.90 | 868.70 | 1793.11 | 3746.48 | 5887.30 | 8654.30 | 11300.36 | 1306 | 1290 | 1490 | 1326 | 1426 | 1446 | 1438 | 1730 |
| STAGATE | 170.58 | – | – | – | – | – | – | – | 15528 | – | – | – | – | – | – | – |
| GraphST | 188.67 | 537.40 | – | – | – | – | – | – | 6114 | 12950 | – | – | – | – | – | – |
| SpaceFlow | 40.64 | 20.56 | 45.86 | 86.15 | 78.71 | 111.64 | 165.97 | 188.44 | 2644 | 3004 | 3826 | 5980 | 10252 | 13998 | 18282 | 21678 |
| SEDR | 23.610 | 48.347 | 147.386 | – | – | – | – | – | 2520 | 5796 | 21458 | – | – | – | – | – |

**Table S3. Runtime and memory usage of STAHD and baseline methods on the Human Tonsil dataset**

|  |
| --- |

| **Dataset** | **Human Breast Cancer（663,857 Spots）** | | | | | | | | | | | | | | | |
| --- | --- | --- | --- | --- | --- | --- | --- | --- | --- | --- | --- | --- | --- | --- | --- | --- |
| Method | Runtime（s） | | | | | | | | Memoryusage（GB） | | | | | | | |
|  | 3% | 5% | 10% | 20% | 40% | 60% | 80% | 100% | 3% | 5% | 10% | 20% | 40% | 60% | 80% | 100% |
| STAHD  (batch_size=256) | 378.52 | 646.34 | 1284.33 | 2733.5 | 6616.33 | 8414.88 | 11960.38 | 13985.14 | 1506 | 1506 | 1734 | 1770 | 1860 | 1660 | 1930 | 1980 |
| STAGATE | 100.96 | – | – | – | – | – | – | – | 14614 | – | – | – | – | – | – | – |
| GraphST | 182.29 | 511.28 | – | – | – | – | – | – | 5924 | 12482 | – | – | – | – | – | – |
| SpaceFlow | 36.84 | 35.60 | 50.48 | 53.21 | 126.46 | 170.15 | 209.84 | 256.27 | 2624 | 2810 | 3782 | 5716 | 10024 | 13687 | 17874 | 21206 |
| SEDR | 23.31 | 45.89 | 143.60 | – | – | – | – | – | 2422 | 5612 | 21000 | – | – | – | – | – |

**Table S4. Runtime and memory usage of STAHD and baseline methods on the Human Breast Cancer dataset.**

**
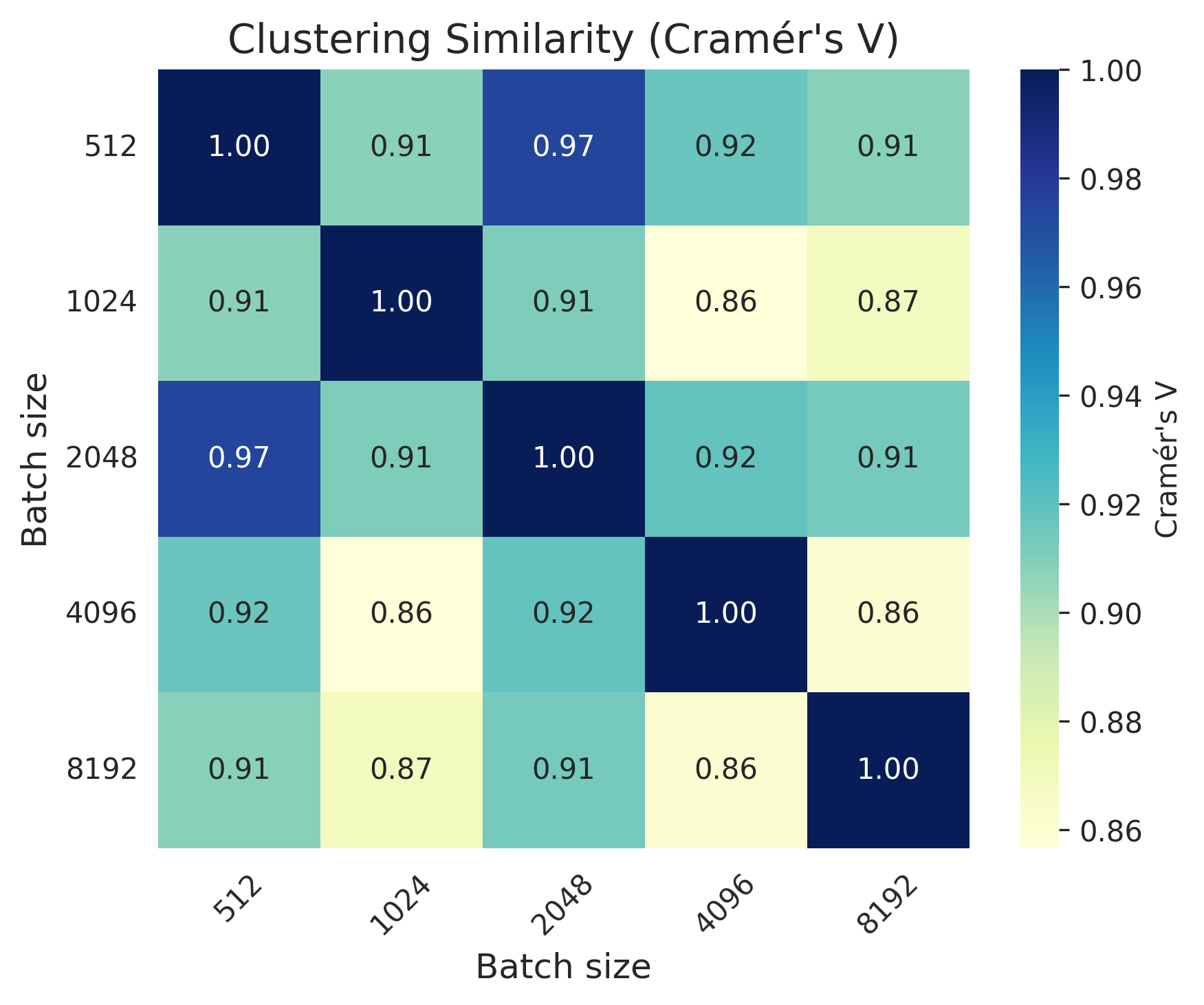
**

**Table S5. Pairwise ARI comparisons between STAHD and other methods (Wilcoxon signed-rank test).**

| **Method** | **p-value** | **n(slices)** | **Significance** |
| --- | --- | --- | --- |
| **STAGATE** | **0.0005** | **12** | ******* |
| **GraphST** | **0.1099** | **12** | **n.s.** |
| **SpaceFlow** | **0.0005** | **12** | ******* |
| **SEDR** | **0.0269** | **12** | ***** |
| **PAST** | **0.093** | **12** | ****** |

**(n.s. = not significant, p < 0.05 = *, p < 0.01 = **, p < 0.001 = ***)**
